# Constitutively active SARM1 variants that induce neuropathy are enriched in ALS patients

**DOI:** 10.1186/s13024-021-00511-x

**Published:** 2022-01-06

**Authors:** A. Joseph Bloom, Xianrong Mao, Amy Strickland, Yo Sasaki, Jeffrey Milbrandt, Aaron DiAntonio

**Affiliations:** 1 Needleman Center for Neurometabolism and Axonal Therapeutics and Department of Genetics, Washington University School of Medicine in Saint Louis, St. Louis, MO USA; 2 Needleman Center for Neurometabolism and Axonal Therapeutics and Department of Developmental Biology, Washington University School of Medicine in Saint Louis, St. Louis, MO USA

**Keywords:** ALS, SARM1, Neurodegeneration, Axon, NAD, Human genetics, Neuropathy

## Abstract

**Background:**

In response to injury, neurons activate a program of organized axon self-destruction initiated by the NAD^+^ hydrolase, SARM1. In healthy neurons SARM1 is autoinhibited, but single amino acid changes can abolish autoinhibition leading to constitutively active SARM1 enzymes that promote degeneration when expressed in cultured neurons.

**Methods:**

To investigate whether naturally occurring human variants might disrupt SARM1 autoinhibition and potentially contribute to risk for neurodegenerative disease, we assayed the enzymatic activity of all 42 rare *SARM1* alleles identified among 8507 amyotrophic lateral sclerosis (ALS) patients and 9671 controls. We then intrathecally injected mice with virus expressing *SARM1* constructs to test the capacity of an ALS-associated constitutively active *SARM1* variant to promote neurodegeneration in vivo.

**Results:**

Twelve out of 42 SARM1 missense variants or small in-frame deletions assayed exhibit constitutive NADase activity, including more than half of those that are unique to the ALS patients or that occur in multiple patients. There is a > 5-fold enrichment of constitutively active variants among patients compared to controls. Expression of constitutively active ALS-associated SARM1 alleles in cultured dorsal root ganglion (DRG) neurons is pro-degenerative and cytotoxic. Intrathecal injection of an AAV expressing the common *SARM1* reference allele is innocuous to mice, but a construct harboring *SARM1*^*V184G*^, the constitutively active variant found most frequently among the ALS patients, causes axon loss, motor dysfunction, and sustained neuroinflammation.

**Conclusions:**

These results implicate rare hypermorphic *SARM1* alleles as candidate genetic risk factors for ALS and other neurodegenerative conditions.

**Supplementary Information:**

The online version contains supplementary material available at 10.1186/s13024-021-00511-x.

## Background

In recent decades, genetic studies have become a principle tool for discovering the molecular pathogenesis underlying neurodegenerative disease. Such investigations have proved especially fruitful for amyotrophic lateral sclerosis (ALS), a rare and devastating motor neuropathy currently linked to over two dozen distinct genetic causes that together explain ~ 10% of ALS cases [1]. As with other diseases, discovery of ALS genes was facilitated by technological innovations enabling successively more powerful methods, from family-based linkage studies to genome-wide association studies, and more recently, large exome-sequencing efforts using sophisticated statistical techniques in large case-control groups [2–4]. However, power to detect such associations is naturally attenuated for genes harboring low penetrance variants that contribute to risk without necessarily causing disease, which is particularly relevant given the oligogenic nature of sporadic ALS [5]. This difficulty is compounded when the functional consequences of rare variants cannot be predicted from sequence alone i.e. for genes where protein-truncating alleles are not assumed to be pathogenic [3], and all the more so when variants in the same gene may confer either increased disease risk or protection. In such cases, the power to detect significant associations can only be improved by systematically characterizing all the functional variants in a candidate gene [6]. Here we take such a hypothesis-driven approach to identify potentially pathogenic alleles of *SARM1*, a key driver of axon degeneration.

Trauma and disease in the nervous system activate an intrinsic axon self-destruction pathway, also known as Wallerian degeneration, which facilitates the orderly clearance of damaged axon segments. This choice between maintaining or actively dismantling axons is primarily determined by the action of SARM1, a TIR-containing NAD^+^ hydrolase that cleaves NAD^+^ to generate nicotinamide and cyclic ADPR (cADPR), a useful biomarker of SARM1 activity [7]. In healthy neurons, SARM1 is maintained in an autoinhibited state, but injury- or disease-induced depletion of the axon survival factor NMNAT2 activates SARM1 leading to a stereotyped sequence of events beginning with rapid loss of NAD^+^, followed by loss of ATP, defects in mitochondrial movement and depolarization, influx of calcium, externalization of phosphatidylserine, and ultimately axon fragmentation [8, 9]. *Sarm1* knockout mice are viable and without apparent phenotypes under routine conditions, but are protected against neurodegeneration in models of axotomy, traumatic brain injury, peripheral neuropathy, glaucoma, and retinal degenerative diseases [10–18]. Conversely, mutations that decrease NMNAT2 activity lead to polyneuropathy in both humans and model organisms [19, 20], suggesting that aberrant SARM1 activation has a role in human disease. Furthermore, the recent observation that single point mutations in *SARM1* can disrupt enzyme autoinhibition [21–25] led us to speculate that naturally-occurring human variants might similarly dysregulate SARM1 and thereby increase disease risk. ALS was an attractive candidate disease because genetic data from multiple large ALS case-control studies are publicly-available. Notably, polymorphisms in the *SARM1* locus have been repeatedly identified by large GWAS of ALS, though the findings were largely discounted due to lack of non-synonymous variants in high linkage disequilibrium and eQTL-effects upon an adjacent gene, *POLDIP2*^26,27^. ALS also warrants particular attention because peripheral axon degeneration accompanies and may precede motoneuron death during ALS progression [26, 27].

To investigate whether *SARM1* mutations that disrupt the enzyme’s autoinhibition are associated with neurogenerative disorders, we sought to identify rare prodegenerative *SARM1* missense variants by biochemically analyzing every rare *SARM1* variant identified in patients and controls from multiple ALS databases. Provocatively, the majority of our strongest candidate variants disrupt SARM1 regulation and confer constitutive activity in vitro. Furthermore, we demonstrate that the enrichment of rare variants found in ALS patients is driven in large part by an enrichment of experimentally-confirmed constitutively active variants. Finally, expression of a constitutively-active *SARM1* allele found in three unrelated patients causes an ALS-like phenotype—motor dysfunction, cell death, axon loss and sustained neuroinflammation—when expressed in the mouse spinal cord via intrathecal delivery. We therefore propose that hypermorphic *SARM1* mutations are a congenital risk factor for ALS. Because the pathogenic effects of SARM1 activity are not limited to motoneurons, we also hypothesize that rare *SARM1* variation may raise risk for other neurodegenerative conditions such as peripheral neuropathies. While this manuscript was under review for publication, a complementary study using different methods also identified constitutive activity in several of the same ALS-associated rare *SARM1* variants reported here [28].

## Materials and methods

### ALS consortia databases

This study is based on anonymized publicly available human genetic data not associated with demographic or clinical information beyond ALS case status. All assayed *SARM1* polymorphisms were reported in one of three databases, accessed January 2020: Project MinE (http://databrowser.projectmine.com), the University of Massachusetts Medical School Sporadic ALS Variant Server (http://als.umassmed.edu/index.php#SALSbrowser), and the ALS Knowledge Portal (http://alskp.org).

### Mice

Male and female WT and *Sarm1* knockout C57BL/6 mice were housed and used under the direction of institutional animal study guidelines at Washington University in St. Louis. The inverted screen test of strength and measurement of grip strength were performed as previously described [29, 30]. All protocols received institutional IACUC approval.

### DRG culture

Mouse DRG culture was performed as previously described [31]. DRG were dissected from embryonic day 13.5 *Sarm1* knockout C57BL/6 or CD1 mouse embryos and cells suspended in growth medium at a concentration of ~ 7 × 10^6^ cells/ml in 96- well tissue culture plates (Corning) coated with poly-d-Lysine (0.1 mg/ml; Sigma) and laminin (3 μg/ml; Invitrogen). Lentiviral particles containing *SARM1* variants and EGFP were generated as previously described [31]. Lentivirus was added (1–10 × 10^3^ pfu) after 1–2 days (DIV) and metabolites were extracted or assays were performed at 6–7 DIV. Cell death was quantified by assaying mitochondrial function (MTT assay), as previously described [32].

### Automated quantification of Annexin V staining

To quantify Annexin V staining, the Alexa Fluor™ 568 conjugate (ThermoFisher) was added to the cultured neurons at a 1:100 dilution four days after viral infection. Bright field and fluorescent images were acquired one hour later using Operetta. Unbiassed image analysis was performed using ImageJ as follows: total axon area was measured from the binary bright field images after subtracting background. For Annexin fluorescent intensity measurement, the fluorescent images were background subtracted and then Annexin positive area was defined using the particle analyzer. Data was reported as the total fluorescent intensity of the Annexin positive area divided by the axon area.

### DRG metabolite extraction and metabolite measurement

At DIV6, tissue culture plates were placed on ice and culture medium replaced with ice-cold saline (0.9% NaCl in water, 500 μl per well). Saline was removed and replaced with 160 μl ice cold 50% MeOH in water. Solution was transferred to tubes containing 50 μl chloroform on ice, shaken vigorously, and centrifuged at 20,000 *g* for 15 min at 4 °C. The clear aqueous phase (140 μl) was transferred into microfuge tubes and lyophilized under vacuum. Lyophilized samples were reconstituted with 5 mM ammonium formate (15 μl), centrifuged (13,000 *g*, 10 min, 4 °C), and 10 μl of clear supernatant was analyzed. NAD^+^ and cADPR were measured using LC-MS/MS as previously described [33].

### AAV constructs and virus injections

AAV particles with a mixture of Php.s and Php.eB capsids [34], containing a human *SARM1* gene construct fused to EGFP, under the control of the human synapsin promoter, were produced by the Viral Vector Core of the Hope Center for Neurological Disorders at Washington University in St. Louis. Viral particles were purified by iodixanol gradient ultracentrifugation and virus titers were measured by dot blot. Under light anesthesia with Avertin, 6 × 10^11^ viral genomes were injected intrathecally at L6/S1. Viral expression in mice 12-weeks post injection was confirmed by detecting EGFP expression via immunohistochemical analysis of sectioned DRGs.

### Immunohistochemistry, imaging and quantification

After perfusion with PBS followed by 4% PFA in PBS, tissues were fixed in 4% PFA in PBS for 1 h at room temperature and placed in 30% sucrose in PBS overnight at 4 °C, then embedded in OCT (Tissue-Tek), frozen on dry ice, and stored at − 80 °C. Longitudinal sections of 6 μm or cross-sections of 20 μm were obtained using a cryostat and slides were stored at − 20 °C. DRG and nerve slides were post-fixed in cold acetone, then washed with PBS. Spinal cord slides were simply washed three times in PBS. All slides were subsequently blocked with 4% BSA and 1% Triton X-100 in PBS and incubated with rat anti-CD68 (1:500; Bio-Rad) and mouse-anti-GFP conjugated to Alexa Fluor 488 (1:250; Thermo Fisher Scientific) overnight in the blocking buffer. Slides were then washed, incubated in secondary antibodies (Jackson ImmunoResearch Laboratories), washed, and mounted in Vectashield with DAPI. Slides were imaged using a DMI 4000B confocal microscope (Leica Microsystems) with a 20× oil objective and DFC 7000-T camera (Leica Microsystems). For quantification, at least four images were measured per animal. CD68-positive cells were counted by a researcher blinded to the images’ treatment group. The total CD68-stained area and nerve area in each image was quantified with the particle analyzer in ImageJ using a uniform threshold.

### TUNEL apoptosis detection

TUNEL was performed as previously described [35]. Slides prepared for immunohistochemistry were thawed then postfixed with 4% PFA for 10 min at room temperature, washed thoroughly with PBS, incubated with 10 μg/ml proteinase K for 15 min at 37 °C, then washed with PBS. A positive control slide was incubated in DNase I (1 U/ml) for 1 h at RT, then washed with PBS. Slides were then pretreated with TdT buffer (25 mm Tris-HCl, 200 mm sodium cacodylate, 0.25 mg/ml BSA, 1 mm cobalt chloride, Roche Diagnostics) at RT for 10 min. To perform end-labeling, TdT buffer was combined with terminal deoxynucleotidyl transferase (Roche Diagnostics, 400 U/slide) and Biotin-16-dUTP (Roche Diagnostics, 4 μm) and added to slides for 1 h at 37 °C. Slides were thoroughly washed with PBS, then blocked for 30 min with 5% normal goat serum in PBS with 0.3% Triton-X, then incubated with Alexa-Fluor-conjugated streptavidin (Jackson ImmunoResearch Laboratories) for 30 min at 37 °C. Slides were washed and mounted in Vectashield with DAPI.

### Toluidine blue staining and axon quantification

Sural, sciatic and tibial nerves were fixed in 3% glutaraldehyde in 0.1 M PBS, processed and imaged as previously described [13]. Micrographs were stitched using Leica software and axons were counted using ImageJ. To determine axon size distribution and G ratios of the sciatic nerve, four nonoverlapping areas per cross section were imaged with a 100× oil objective of a Zeiss Axioskop and photographed with a Hitachi camera. Photographs were analyzed using a previously described binary imaging analysis method [36].

### Statistical analysis

The frequency of constitutively active *SARM1* variants in ALS cases vs. controls was compared using Fisher’s Exact test. All other comparisons (metabolite measurements, axon density and size, g-ratio, MTT assay, Annexin-V staining, inverted screen test, grip strength, CD68 staining) were made using two-tailed t-tests. All statistics were calculated using the R software package. All data is available upon request.

## Results

### Enrichment of rare *SARM1* variants in ALS patients

The discovery of single point mutations in *SARM1* that confer constitutive NADase activity leading to degeneration of cultured neurons [10, 22, 23, 37] prompted us to postulate that rare human gain-of-function *SARM1* variants might increase risk for neurodegenerative disease. ALS was of particular interest because of the large-scale axon loss that occurs over the course of the disease [26, 27]. To explore this hypothesis, we chose to investigate *SARM1* variants found in ALS patients, a population where an abundance of whole exome and genome data is available.

We identified a total of 30 rare *SARM1* coding variants (missense and small in-frame deletions with allele frequencies < 0.01% in all gnomAD populations [38]) occurring exclusively in ALS patients, culled from three large publicly-accessible ALS consortia databases that include 8507 cases in total as of January 2020^41–43^ (Table 1). All subjects harboring rare *SARM1* variants are heterozygotes. Despite being extremely rare, eight of these variants were each found in two or three ALS patients. We further identified twelve rare *SARM1* coding variants that occur in control individuals from these databases. Altogether, rare variants occur 45 times in 8507 ALS cases and 15 times in 9671 controls (Table 1), representing a 3.4-fold enrichment of rare *SARM1* variants in ALS patients.

**Table 1:** Rare *SARM1* missense variants and in-frame deletions found in ALS patients

Variant	rsID	Percent Minor Allele Frequency^**a**^			Number of occurrences	
		African	East Asian	European^b^	ALS patients	Controls
**Constitutively active**						
Δ226–232	rs782325355	0	0	0.01	2^d^	0
Δ249–252		0	0	0	1^d^	0
V184G	rs71373646	0.007	0.006	0	3^e^	0
G206S	rs1555585199	0	0	0	2^e^	0
L223P		0	0	0	1^d^	0
R267W	rs11652384	0	0	0.001	1^e^	0
V331E	rs1555585331	0	0	0	1^d^	0
E340K	rs781854217	0	0	0.003	1^d^	0
C482Y		0	0	0	0	1^d^
T385A		0	0	0	1^d^	0
T502P	rs782421919	0	0	0.006	2^d,f^	0
E693D	rs782331635	0	0	0.005	0	2^f^
**Not constitutively active**						
V112I	rs1032963037	0	0	0^c^	1^d^	0
A240E	rs1449836804	0	0	0.004	1^d^	0
R244S		0	0	0	1^d^	0
A250T	rs1555585243	0	0.06	0	1^d^	0
A275V	rs376587698	0	0	0.006	1^d^	0
R310H	rs369186722	0	0	0.01	1^d^	0
A341V	rs373458416	0	0	0.01	2^d^	0
R403P	rs782706244	0	0	0.0009	1^f^	0
Y429F		0	0	0	0	1^d^
E431G	rs1555585662	0	0	0.001	1^d^	0
R465T		0	0	0	1^d^	0
N478S	rs1555585804	0	0	0.001	0	1^f^
D483E	rs377210302	0	0	0.002	0	1^f^
R484C	rs1555585809	0	0	0.0009	1^d^	0
A488E	rs782228906	0.004	0	0.02	2^d^	0
V518L	rs782106973	0	0	0^c^	3^d^	0
S558N		0	0	0	0	1^d^
R569C	rs571724138	0	0	0.005	1^d^	0
R570Q	rs539229444	0	0.008	0.005	1^e^	0
I593T	rs782196205	0	0.005	0.002	0	1^f^
E604K	rs782398426	0.02	0.01	0.005	0	1^f^
M612V	rs782321764	0	0	0.004	0	2^f^
R615H	rs782753946	0.008	0	0.004	2^f^	1^d^
D637Y	rs1451417529	0	0	0	1^d^	0
A646S	rs782676389	0	0	0.0008	1^d^	0
V654M	rs782225125	0	0	0.002	0	2^f^
M672V	rs782774927	0	0	0.004	2^e,f^	0
S684F	rs782256561	0.004	0	0.004	1^f^	0
R697C	rs372946020	0.004	0	0.003	3^f^	1^f^
R702C	rs781850558	0	0.005	0.002	1^f^	0

^a^gnomAD v2

^b^non-Finnish

^c^V518L = 0.009% and V112I = 0.001% in gnomAD v3 non-Finnish Europeans

^d^MinE [37]

^e^ALS Variant Server [39]

^f^ALS Knowledge Portal [38]

### *SARM1* variants found in ALS patients exhibit constitutive NAD^+^ hydrolase activity

**Fig. 1:**
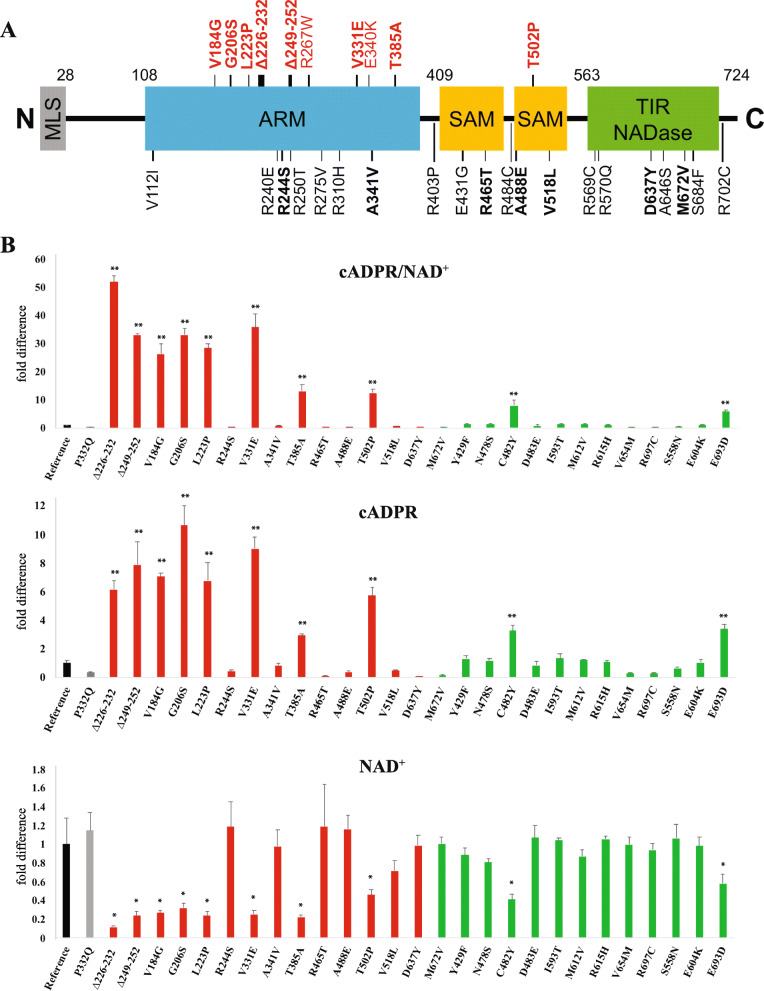
Identification of dysregulated *SARM1* variants found in ALS patients. (**A**) Schematic representation of the domain structure of SARM1 marked with every rare variant found in ALS patients. Constitutively-active variants are indicated above in red. Bold variants were prioritized because they were identified in multiple ALS patients or were unique to ALS patients. Δ indicates an in-frame deletion. MLS, mitochondrial localization signal; ARM, HEAT/Armadillo motif; SAM, sterile alpha motif; TIR, Toll/interleukin-1 receptor homology domain. **(B)** The ratio of cADPR/NAD^+^, cADPR, and NAD^+^ levels from cultured *Sarm1*^*−/−*^ DRG neurons infected with human *SARM1* constructs carrying every rare variants identified in multiple ALS patients or unique to ALS patients (red), every rare variant found in controls (green), and the most common *SARM1* variant, P332Q (gray), relative to the common reference human genome allele of *SARM1* (black). *p < 0.05; **p < 0.0005 difference from reference allele. We demonstrate that strongly constitutively active variants predominate among those *SARM1* variants identified in multiple ALS patients or unique to ALS patients, while the only two significantly active variants discovered in controls are comparatively weak

To investigate whether the rare *SARM1* variants in ALS patients disrupt autoinhibition, we assayed the NAD^+^ hydrolase activities of the mutant enzymes encoded by these variants. We prioritized the variants and first tested a) those identified in multiple ALS patients but not in healthy controls and b) those unique to ALS patients (i.e. not reported in any prior human study as of January 2020). These 15 *SARM1* variants (Fig. 1A, Table 1) account for 51% (20/39) of rare *SARM1* variants in ALS patients genotyped in the three large ALS databases we investigated. To examine the properties of these mutants, we tested them in *Sarm1*^*−/−*^ mouse dorsal root ganglion DRG neurons. We prepared lentiviruses for the 15 *SARM1* mutant constructs, infected *Sarm1*^*−/−*^ neurons, and assessed their NAD^+^ hydrolase activity. As validated in our prior publication [21], the ratio of the SARM1-specific biomarker cADPR [33] over its parent metabolite NAD^+^ is the best measure of relative SARM1 activity because cADPR production is specific to SARM1, while NAD^+^ concentration is affected by other cellular processes. Moreover, because NAD^+^ is the limiting substrate, the ratio of the two molecules accounts for both SARM1 enzymatic activity and substrate depletion. Measuring cADPR/NAD^+^, eight of these variants were determined to be constitutively active, i.e. their baseline levels of NAD^+^ were significantly decreased and levels of cADPR and the ratio of cADPR/NAD^+^ were significantly increased in neurons expressing these mutant constructs (Fig. 1). Tellingly, all but one of these constitutively active variants reside in the autoinhibitory ARM domain. By contrast, *SARM1*^P332Q^, the only relatively common variant found in any gnomAD population (1.1% in Europeans) is not constitutively active (Fig. 1). We confirmed that the measured differences in NAD^+^ hydrolase activity were not due to differences in construct expression levels using expression of the EGFP also included in the construct (Additional Files 1 and 2). Indeed, the strongest constitutively active mutant constructs were also associated with the weakest EGFP expression (Fig. 1 and Additional Files 1 and 2), a phenomenon we and others observe routinely [28], which we attribute to reduced protein translation due to NAD^+^ depletion in the infected cells. Thus, we are likely underestimating the relative activity of constitutively active alleles. We also saw similar activity when mutant *SARM1* constructs were expressed in wildtype mouse DRG neurons (i.e. in the presence of endogenous wildtype SARM1) (Additional File 3).

Encouraged by these results, we assayed the activities of an additional 15 rare missense variants. These were considered poorer candidates because each is observed in only a single ALS patient and they are not unique to the patients as they are also found in the gnomAD database. Among these, we identified two additional constitutively active variants (Table 1). In total, 40% (4/10) of the SARM1 variants with constitutive NAD^+^ hydrolase activity occur in multiple ALS patients.

### Enrichment of constitutively-active *SARM1* variants in ALS patients

In order to determine whether constitutively active *SARM1* variants occur significantly more frequently among ALS patients, we further assayed the activities of all twelve rare *SARM1* missense variants reported among 9671 healthy control individuals included in the large publicly-accessible ALS consortia databases (Table 1). Among these, two variants (one of which occurred twice) demonstrated significantly greater enzymatic activity than the reference allele of SARM1, although notably they are the weakest variants that we define as constitutively active (see cADPR/NAD^+^ ratio in Fig. 1). In total, 3 of 9671 controls versus 15 of 8507 cases carry a constitutively active *SARM1* variant, representing a significant 5.7-fold enrichment among cases (Fisher’s Exact *p* = 0.0032).

### Constitutively active *SARM1* variants found in ALS patients promote degeneration of cultured neurons

**Fig. 2:**
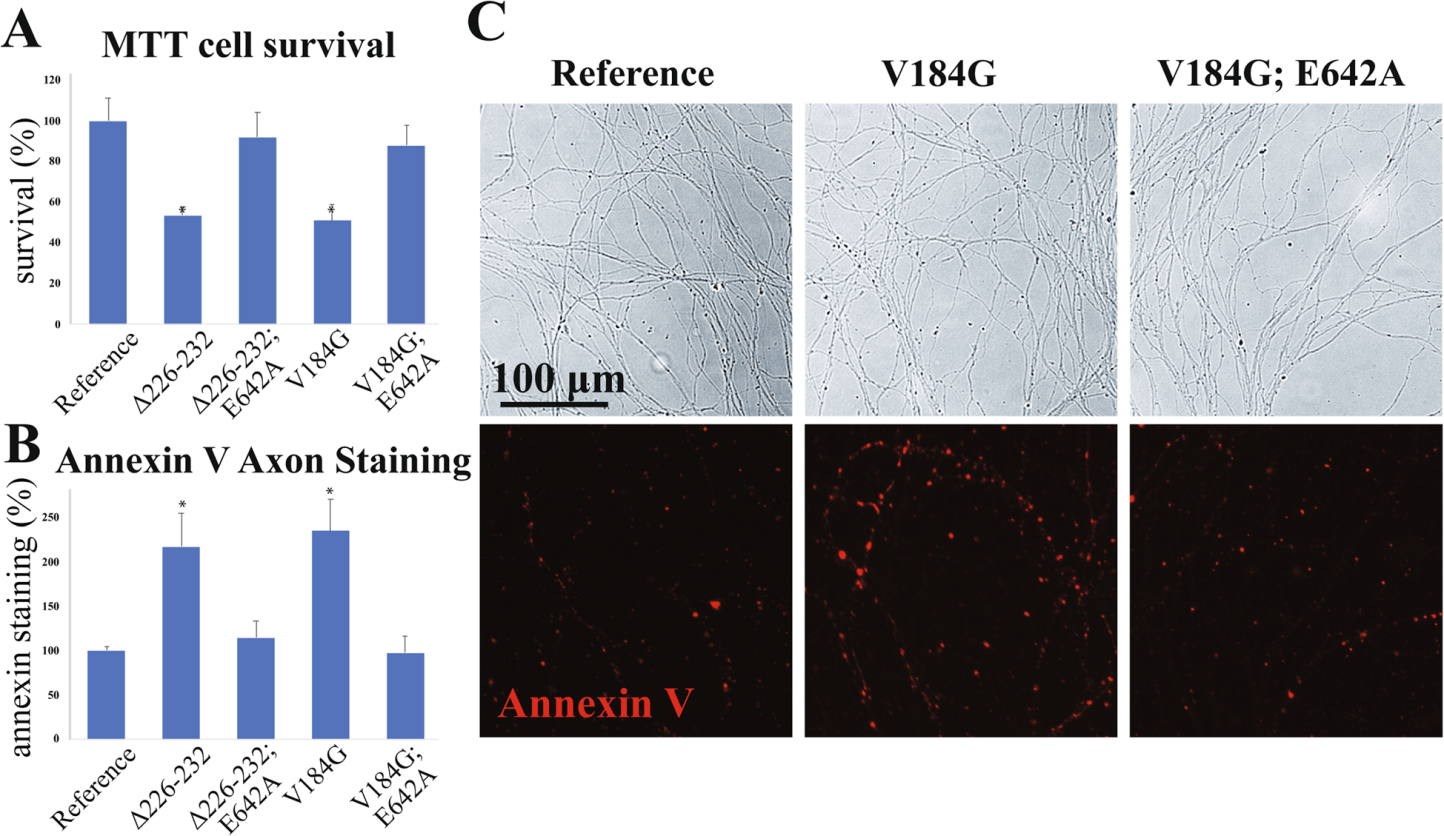
Neurodegeneration in cultured neurons transfected with ALS-associated *SARM1* variants. (**A**) Neuron death as measured by the MTT assay and (**B**) axon degeneration as measured by Annexin V staining in *Sarm1*^*−/−*^ DRG neurons infected with lentivirus expressing *SARM1* variant constructs as well as double mutant constructs including E642A, a point mutation that disrupts SARM1 NAD^+^ hydrolase activity, relative to the common *SARM1* reference allele. All data are expressed as the percent of surviving cells or area of Annexin stained axons relative to neurons infected with the reference allele. (**C**) Representative bright-field and Annexin V-stained images of axons from *Sarm1*^*−/−*^ DRG cultures infected with variant and *SARM1* reference allele constructs. *p < 0.005 difference from reference allele. These results demonstrate that constitutive SARM1 activity causes cell death and axon degeneration while expression of control SARM1 does not

The structure of SARM1 in its autoinhibited state, as determined by cryo-EM, indicates the protein exists as a compact octamer with its TIR NADase domains isolated from one another, preventing the TIR oligomerization necessary for enzymatic activation. Mutational analyses identified five distinct and non-redundant interfaces required for autoinhibition that support both intramolecular and intermolecular interactions [24]. Point mutants that disrupt any of these interfaces result in dysregulation of SARM1 activity and this constitutive SARM1 activity promotes the degeneration of cultured neurons [10, 22, 23, 37]. As in those prior studies, we also recognized changes in cell morphology consistent with cell death in *Sarm1*^*−/−*^ mouse DRG neurons expressing constitutively active variants and chose to quantify cell death and degeneration for two of the variants, *SARM1*^*V184G*^ and *SARM1*^*Δ226–232*^. These two variants were chosen to assay because they occurred in three and two independent ALS patients respectively (Table 1). The mutant enzymes were expressed in *Sarm1*^*−/−*^ DRG neurons and degeneration was measured by two methods. Fluorescently-labeled Annexin V, which binds to phosphatidylserine, was used to determine whether the expression of either variant construct significantly compromises axon health. Annexin V binding is a useful proxy for axon health as neurites undergoing Wallerian degeneration expose phosphatidylserine on their extracellular surfaces similarly to apoptotic cells [9, 40]. Neuronal death was quantified using an oxidoreductase activity assay, a common measure of cell viability. Both assays demonstrated that both ALS-associated *SARM1* variants produced a significant degenerative effect relative to the common reference allele (Fig. 2).

While these variants exhibit constitutive NAD^+^ hydrolase activity, it is formally possible that they mediate their pro-degenerative effects via a distinct toxic mechanism. To investigate this alternate hypothesis, we generated constructs containing two mutations, either of the ALS-associated variants, i.e. *SARM1*^*V184G*^ or *SARM1*^*Δ226–232*^, together with E642A, a point mutation in the TIR domain that disrupts the catalytic glutamate required for SARM1 NAD^+^ hydrolase activity and axon degeneration [23, 41–44]. In both cases, introducing E642A abolishes enzymatic activity and the detrimental effects of the constructs on cell body and axon health (Fig. 2). Hence, these ALS patient-derived *SARM1* variants promote degeneration via loss of autoinhibition and resulting constitutive NAD^+^ hydrolase activity.

### The ALS-associated *SARM1*^*V184G*^ variant induces motor impairment, peripheral axon loss and neuroinflammation in mice

To test whether rare ALS-associated *SARM1* variants can promote neurodegeneration in vivo, AAV viral vectors were administered intrathecally to male and female six-week old wild-type mice, expressing either the common human allele of *SARM1* (the reference allele) or *SARM1*^*V184G*^. V184G was chosen to examine in vivo because it was the rare variant that occurred most frequently among ALS patients, found in three independent subjects. In these constructs, each SARM1 protein was fused to EGFP and expressed under the control of the human synapsin promoter. AAV viruses were produced using a mixture of PHP. S and PHP.eB serotype capsids (both derived from AAV9 [34]) in order to infect neurons in the spinal cord and DRGs.

**Fig. 3:**
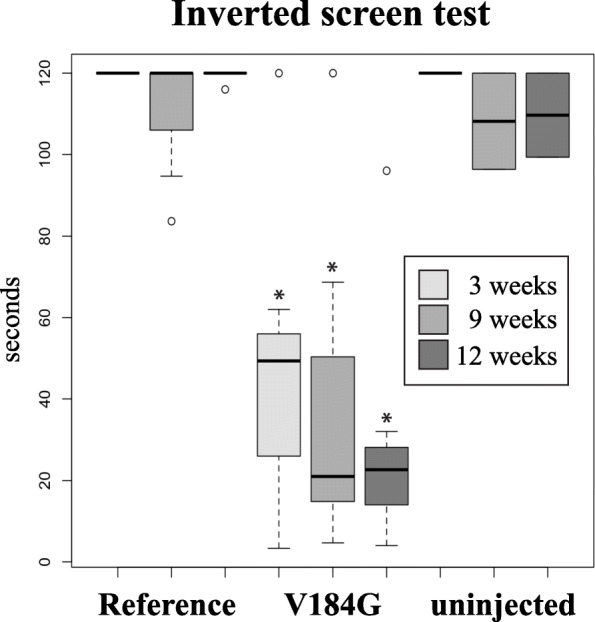
Motor dysfunction in mice injected intrathecally with a *SARM1*^*V184G*^ AAV construct. Average time suspended from an inverted screen (maximum 120 s) for C57BL/6 mice injected with a human *SARM1* reference allele (*n* = 8) or *SARM1*^*V184G*^ (*n* = 7) AAV compared to uninjected controls (*n* = 3) 3, 9 and 12 weeks post-injection. *p < 0.005 difference from both the reference allele and uninjected controls. These results demonstrate that infection with *SARM1*^*V184G*^ causes persistent motor dysfunction while the control *SARM1* construct does not

Animals injected with AAV expressing the common *SARM1* allele had no discernible behavioral phenotypes. By contrast, those injected with AAV-*SARM1*^*V184G*^ exhibited motor impairment. Two of the mice rapidly progressed to full limb paralysis 3–4 days after injection. Other animals injected with *SARM1*^*V184G*^ (7/9) displayed less dramatic motor deficits characterized by significant muscle weakness as measured by the inverted screen assay (Fig. 3) or hindlimb grip strength (Additional File 4). These deficits were detected within 3 weeks of injection and did not progress significantly during the 12-week observation period.

**Fig. 4:**
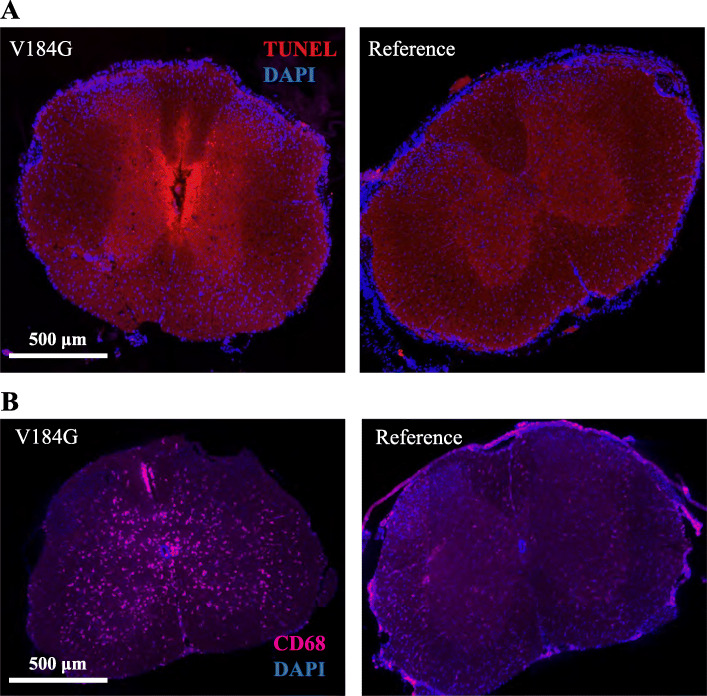
Rapid cell death and neuroinflammation in mice injected intrathecally with a *SARM1*^*V184G*^ AAV construct. This figure demonstrates anatomical findings from the small subset of mice with rapid onset pathology. (**A**) Representative images of spinal cord sections stained with DAPI and the apoptosis marker TUNEL from mice 2 days after injection with a *SARM1*^*V184G*^ or *SARM1* human reference allele construct. (**B**) Representative images of spinal cord stained with DAPI and the macrophage marker anti-CD68 from mice 2 days after injection with a *SARM1*^*V184G*^ or reference allele construct. These images demonstrate that mice that become paralyzed shortly after injection with *SARM1*^*V184G*^ exhibit cell death and activated macrophages in their spinal cords while mice injected with the control *SARM1* construct do not

To characterize the neurodegeneration caused by *SARM*^*V184G*^ expression, the intrathecally injected mice were examined for evidence of axon degeneration and neuron loss. We examined the two mice that became rapidly paralyzed and the other mice with less severe disease as separate cohorts because of the difference in phenotype. Unfortunately, because we do not see EGFP expression with any construct until at least a week post-infection, we were unable to use EGFP to confirm expression in mice sacrificed early. In the spinal cords of the paralyzed mice, there was clear evidence of cell death around the ependymal canal as detected by TUNEL staining (Fig. 4, Additional File 5). Neuroinflammation was observed throughout the spinal cord of these mice as evidenced by prevalent staining for CD68, a marker of activated macrophages (Fig. 4). Neither of these phenotypes were observed in animals injected with the common *SARM1* allele construct (Fig. 4). In this cohort, levels of CD68 staining in sciatic nerves did not differ between mice injected with *SARM1*^*V184G*^ and the control *SARM1* AAV (Additional File 5), nor did any of the mice display significant myelin defects or vacuolization in the sural, sciatic or tibial nerves 3–4 days post-infection (data not shown).

**Fig. 5:**
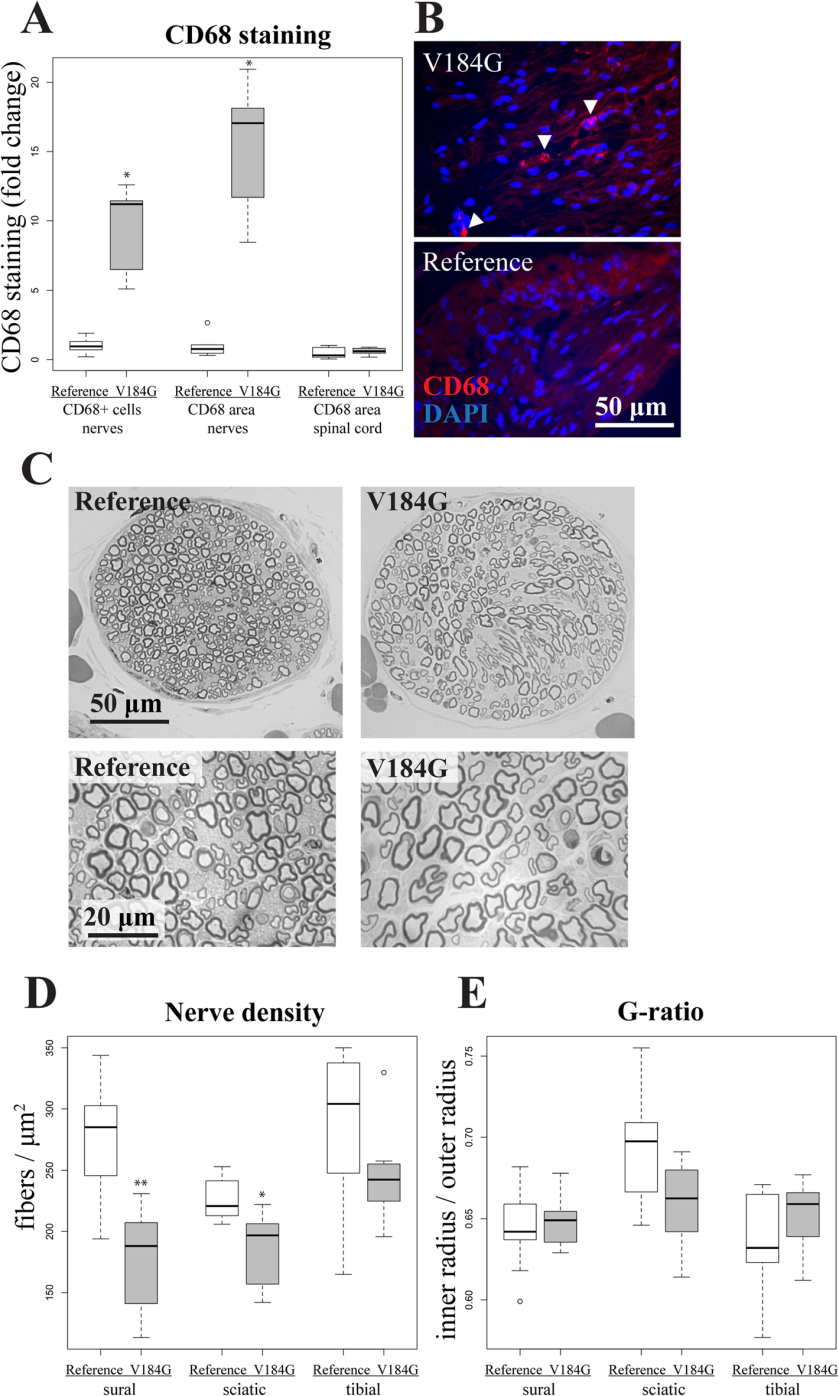
Persistent neuroinflammation and axon loss in mice injected intrathecally with a *SARM1*^*V184G*^ AAV construct. This figure demonstrates anatomical findings from the larger subset of mice with slower onset pathology. (**A**) The normalized average number of cells stained by the macrophage marker anti-CD68 in nerve, and the average percent area of total anti-CD68 staining in nerve and in spinal cord sections, from C57BL/6 mice injected with a *SARM1*^*V184G*^ (*n* = 7) AAV construct relative to those injected with a human *SARM1* reference allele construct (*n* = 8) 12 weeks post-injection; *p < 10^− 4^ difference from reference allele. (**B**) Representative images of sural nerve stained with DAPI and anti-CD68 from mice 12 weeks after injection with a *SARM1*^*V184G*^ or reference allele construct. White arrows indicate CD68-positive activated macrophages. (**C**) Representative images of toluidine blue stained sural nerve sections. (**D**) Average fibers per cross-sectional μm^2^ in sural, sciatic and tibial nerves from mice 12 weeks after injection with a *SARM1*^*V184G*^ (*n* = 7 mice) or reference allele construct (*n* = 8); *p < 0.05, **p < 0.001. (**E**) Average g-ratio (ratio between the inner and outer myelin sheath) for axons in the sural, sciatic and tibial nerves from mice 12 weeks after injection with a *SARM1*^*V184G*^ or reference allele construct. These analyses demonstrate that mice injected with *SARM1*^*V184G*^ exhibit axon loss and persistent neuroinflammation in their nerves 12 weeks after infection, compared to mice injected with a control *SARM1* construct

The mice treated with *SARM1*^*V184G*^ that displayed a less severe behavioral response were sacrificed twelve weeks post-injection. Expression of viral constructs in these mice was confirmed using the presence of EGFP in the DRGs and were similar for each construct (Additional Files 6 and 7). Pathological inspection of their spinal cords revealed no evidence of ongoing apoptosis or elevated CD68 staining (Fig. 5). Their peripheral nerves, however, contain almost 10-fold more CD68-positive macrophages than those treated with the control *SARM1* allele (Fig. 5). Macrophages increase in size upon activation [45], and the *SARM1*^*V184G*^-infected mice also have a 1.6-fold greater CD68-stained area per cell than do control mice, yielding a 15.2-fold difference in total CD68-stained area. Hence, neuronal expression of *SARM1*^*V184G*^ triggers an elevated inflammatory response in peripheral nerves that persists for at least twelve weeks after treatment [46].

The average fiber densities in the peripheral nerves of the *SARM1*^*V184G*^-injected mice are also lower than those injected with AAV expressing the common human allele, demonstrating that expression of the constitutively active SARM1 promotes axon loss. The number of axons were counted in the sural, sciatic and tibial nerves. In both the sural and sciatic nerves, the density of axons is significantly lower (*p* < 0.001), a trend that is evident in the tibial nerve but that did not reach statistical significance (Fig. 5). The average axon size and extent of myelination (g-ratio) does not differ significantly between the variant and common *SARM1* allele treated animals (Fig. 5, *p* > 0.1, *n* = 16). Myelin defects and vacuolization are not observed in these nerves, indicating a lack of ongoing axon loss. The lack of axon defects at twelve weeks is consistent with the early, but stable, deficits in motor function observed in mice receiving the *SARM1*^*V184G*^ virus (Fig. 3). We interpret these data as evidence that a subset of neurons—those infected with virus and sufficiently susceptible to SARM1-dependent degeneration—lost their axons before three weeks, while others, including uninfected neurons, remained healthy and functional up to twelve weeks. Inter-animal differences in motor dysfunction severity likely reflect variability in infection efficiency.

## Discussion

SARM1, the chief axon executioner, directs the ordered elimination of damaged axons, a process called Wallerian degeneration. In healthy axons, this potent NAD^+^ hydrolase is maintained in an autoinhibited state, waiting to be activated by injury or disease. SARM1 activation leads to a rapid loss of NAD^+^, metabolic catastrophe and subsequent axon fragmentation [7, 9]. Due to its central role in programmed axon destruction, prior investigations of SARM1 vis-à-vis disease have focused on inhibiting the pathway to block degeneration downstream of specific injury and disease processes [10–16]. This is the first study to address genetically-determined gain-of-function SARM1 activity as a candidate disease mechanism in itself, building upon the discovery of engineered mutations that disrupt SARM1 autoinhibition and promote neurodegeneration in vitro [21–25]. Provocatively, we find that many rare *SARM1* variants found in ALS patients also lack normal autoinhibition, and that such an allele induces neurodegeneration and neuroinflammation when expressed in the mouse nervous system.

A number of prior findings are consistent with the hypothesis that SARM1 activity contributes to pathological neurodegeneration, particularly in ALS. First, non-coding variants in the *SARM1* locus were repeatedly detected in genome-wide association studies of ALS, though a causal link has been largely discounted [47, 48]. Second, aggregation of TDP-43, a hallmark of most ALS (excluding *SOD1*-ALS), results in the reduced expression of *STMN2*, a key axon survival factor that inhibits SARM1-mediated axon destruction [49–52]. Finally, *Sarm1* loss suppresses neurodegenerative phenotypes in many injury and disease models [10–18], including a mouse that expresses pathogenic human TDP-43 [53]. However, this suppression does have limits, as *Sarm1* deletion does not ameliorate ALS symptoms in the *SOD1*^*G93A*^ mouse model [30].

The hypothesis that naturally-occurring *SARM1* polymorphisms could disinhibit normal enzyme activity and predispose to degenerative disease led us to examine exome sequence data from several well-annotated studies of ALS [39, 54, 55]. We discovered multiple rare *SARM1* variants in these patients that result in constitutive activity due to loss of autoinhibition, and consequently promote neuron death and axon degeneration *in vitro*. Importantly, a single point-mutation that disrupts SARM1 catalytic activity is sufficient to negate the pro-degenerative effects of these ALS-associated variants, demonstrating the degenerative phenotype requires SARM1 NAD^+^ hydrolase activity and is not due to a non-specific mechanism such as the toxic aggregation of a misfolded protein. Expression in the mouse CNS of a *SARM1* construct harboring one such variant causes motor dysfunction and sustained neuroinflammation, abnormalities not observed in mice treated with the common human reference allele of *SARM1*. The mechanism by which constitutive activity would predispose to neurodegeneration appears straightforward. Low NAD^+^ is a death sentence for energy-hungry neurons and is associated with both disease and aging-related functional defects [56, 57]. Indeed, NAD^+^ augmentation ameliorated ALS symptoms in a small clinical trial [58]. We speculate that the contrast between virus-infected mice that rapidly display severe degenerative phenotypes, and human ALS patients who are typically diagnosed only after several decades of life, likely reflects the difference in SARM1 expression—i.e. viral over-expression precipitates abrupt metabolic catastrophe in this model, whereas chronic suboptimal NAD^+^ levels lead to gradual motoneuron attrition in patients. Hence, our studies demonstrate what activating *SARM1* mutations can do in an animal model, but assessing whether such mutations model ALS will require generation of germline mutations in *Sarm1* that mimic the human variants.

Sophisticated statistical methods and next-generation sequencing have allowed the contribution of rare variation to disease risk to be addressed using techniques that identify genes containing an excess of presumably deleterious rare variants among cases relative to healthy controls [4]. However, because SARM1 is a switch that may be pushed either ‘off’ or ‘on’, the consequence of any genetic perturbation defies simple assumptions. Extensive structure/function analysis has shown that single point mutations in *SARM1* can 1) abolish NADase function [41], 2) create a neuroprotective dominant negative [59], 3) alter the enzyme’s sensitivity to regulation by NMN and NAD^+^ [21, 22], or 4) disrupt its autoinhibitory domain leading to pro-degenerative constitutive activity [24]. Indeed, altering a specific residue by substituting different amino acids at the same position can confer opposite effects [21], underscoring the futility of attempting to determine function in silico rather than experimentally.

Typically, human geneticists discover genotype-phenotype associations based on assumptions about the functional consequences of genetic variation, and laboratory biologists pursue the mechanisms underlying these associations using relevant experimental systems. But there are important limitations of this paradigm, including 1) that small and under-studied populations are disadvantaged because findings from the best-powered association studies (usually of European-ancestry subjects) are inevitably prioritized, 2) genetic diversity can hamper discovery because associations are most easily found in homogenous populations where risk is driven by a few common variants rather than many less-frequent variants [60–65] and 3) initial association tests fail to account for the unpredictable consequences of most human mutations. This is especially true when protein-truncating variants are not pathogenic, and when gain-of-function and loss-of-function variants in the same gene have opposite effects on disease risk [6]. By comprehensively characterizing the functional consequences of all potentially pathogenic variants in a strong candidate locus, we sought to reverse the usual paradigm and increase our power to detect an association based on a strong mechanism-based hypothesis. The result was to demonstrate a significant enrichment among ALS patients of a particular *class* of variants in our candidate gene—those conferring constitutive SARM1 enzymatic activity. In addition, with the increasing use of exome sequencing for genetic diagnosis of neurodegenerative disease [66], our specific results provide immediately clinically relevant information, as well as provide a methodology for assessing the likely pathogenicity of further *SARM1* variants in patients. Our results regarding naturally-occurring human variants also align closely with the findings of prior structure-function investigations of SARM1, namely the centrality of the ARM domain in SARM1 autoinhibition [21–25]. All but three of the constitutively active variants we identified lie in the ARM domain, and these exceptions are the three weakest constitutively active alleles assayed (Fig. 1). The two weakest variants were also those found in healthy controls, and we speculate that the quantitative difference between these and other variants may suggest a threshold between significant differences in in vitro activity and clinically significant differences in pathogenicity. While both of these control variants show at least 5-fold higher basal enzymatic activity than the common reference allele of SARM1 in vitro, the most common ALS patient-derived variant, V184G, has more than 26-fold higher activity (Fig. 1). Finally, we also observe that constitutively active variants do not fully account for the enrichment of rare *SARM1* variants associated with ALS. While constitutively-active variants are 5.7-fold enriched, non-constitutively active rare variants are also 2.8-fold enriched in ALS patients compared to controls. Notably, our group has previously probed the allosteric modulation of SARM1 using engineered mutations that alter SARM1 activation without changing basal activity [21]. We therefore hypothesize that other classes of pathogenic SARM1 variants may exist that act via mechanisms other than directly disrupting enzyme autoinhibition.

Fortunately, though the number of ways to disrupt SARM1 may be myriad, for patients harboring deleterious *SARM1* polymorphisms the therapeutic solution could be universal. As we have demonstrated, NAD^+^ hydrolase activity is required for the prodegenerative function of SARM1 [41], and that catalytic machinery is an attractive drug target. Indeed, small molecule SARM1 inhibitors are currently in development [67], and we have shown that a *SARM1* dominant negative gene therapy can potently block SARM1-mediated programmed axon degeneration in mice [59]. Establishing that SARM1 inhibition is safe and effective in carriers of pathogenic *SARM1* variants could provide a vital stepping stone toward the use of SARM1-directed therapeutics more generally for ALS and other diseases that involve axon degeneration.

## Conclusions

In summary, we identified 12 constitutively active SARM1 variants among all 42 rare missense variants reported among 8507 ALS patients and 9671 controls. These include more than half of those that are unique to those patients or that occur in multiple patients. We also demonstrate a > 5-fold enrichment of constitutively active variants among patients compared to controls. Expression of constitutively active ALS-associated *SARM1* alleles is pro-degenerative, both in vitro in cultured neurons and in vivo in the mouse central nervous system. These results implicate rare hypermorphic *SARM1* alleles as candidate genetic risk factors for ALS and other neurodegenerative conditions.

## Supplementary Information

**Additional file 1. **Bright field and matching fluorescent images of cultured mouse DRG neurons infected with *SARM1*-EGFP constructs including the human reference allele, every constitutively active SARM1 variant found in ALS patients and ten rare SARM1 variants found in controls. The expression of EGFP in neurons infected with ALS-associated constitutively active SARM1 variants is similar or less than EGFP expression in reference SARM1-infected neurons (compare background fluorescence to cell bodies).

**Additional file 2. **EGFP fluorescence intensity per cell area in cultured mouse DRG neurons infected with *SARM1*-EGFP constructs including every constitutively active SARM1 variant found in ALS patients and ten rare SARM1 variants found in controls, normalized to the human reference *SARM1* allele. Reduced EGFP expression in cells infected with constitutively active SARM1 alleles is likely due to NAD^+^ depletion by SARM1 leading to impaired protein translation.

**Additional file 3. **The ratio of cADPR/NAD^+^ levels in cultured CD1 wild-type DRG neurons (i.e. expressing endogenous SARM1) infected with human *SARM1* constructs carrying rare variants identified in ALS patients. The variants are significantly more active than the reference human SARM1 allele in the presence of endogenous SARM1. This activity is dependent on the activity of the SARM1 mutant, as demonstrated by the loss of activity when a second activity-abolishing mutation, E642A, is introduced into the construct. Data are expressed relative to cADPR/NAD^+^ in reference SARM1-expressing neurons.

**Additional file 4.** Forelimb grip strength in male and female mice 3 weeks after injection with SARM1 AAV constructs.

**Additional file 5.** Percent area of TUNEL staining in spinal cord, and percent area of CD68 staining in sciatic nerves, two days after injecting mice with SARM1 AAV constructs.

**Additional file 6. **Representative images of dorsal root ganglia from mice 12 weeks post intrathecal injection with AAV constructs containing *SARM1* or the ALS-associated constitutively active variant *SARM1*^*V184G*^ fused to *EGFP*, demonstrating similar expression levels from the two constructs.

**Additional file 7. **Percent of EGFP positive dorsal root ganglia from mice intrathecally injected with AAV constructs containing the reference *SARM1* allele or *SARM1*^*V184G*^ fused to *EGFP*.

## Data Availability

All data relevant to this study are contained within the article.

## Abbreviations

**AAV**: Adeno-associated virus
**ALS**: Amyotrophic Lateral Sclerosis
**ARM**: HEAT/Armadillo motif
**cADPR**: Cyclic adenosine diphosphate ribose
**CD68**: Cluster of Differentiation 68
**DRG**: Dorsal root ganglion
**EGFP**: Enhanced green fluorescent protein
**MLS**: Mitochondrial localization signal
**MTT**: 3-(4,5-dimethylthiazol-2-yl)-2,5-diphenyltetrazolium bromide
**NAD**: Nicotinamide adenine dinucleotide
**NMNAT2**: Nicotinamide mononucleotide adenylyltransferase 2
**TIR**: Toll/Interleukin receptor
**TUNEL**: Terminal deoxynucleotidyl transferase dUTP nick end labeling
**SAM**: Sterile alpha motif
**SARM1**: Sterile alpha and TIR motif containing 1
