# Supplementary figures and images for: Constitutively active SARM1 variants that induce neuropathy are enriched in ALS patients
Source: Mol Neurodegener. 2022 Jan 6;17:1. doi: 10.1186/s13024-021-00511-x (PMC8739729; doi:10.1186/s13024-021-00511-x)

**Constitutively-active ALS patient variants**

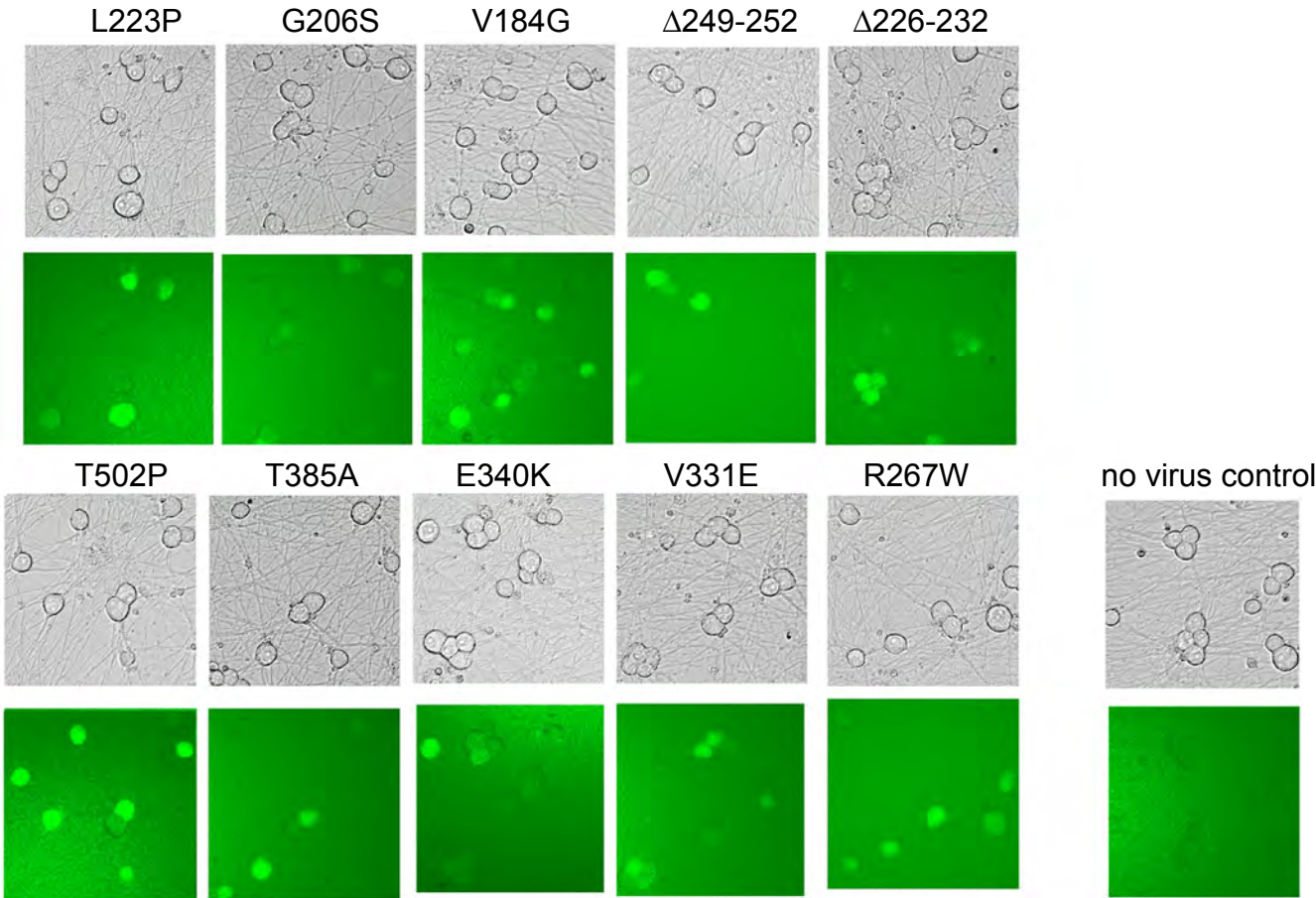

**Control variants**

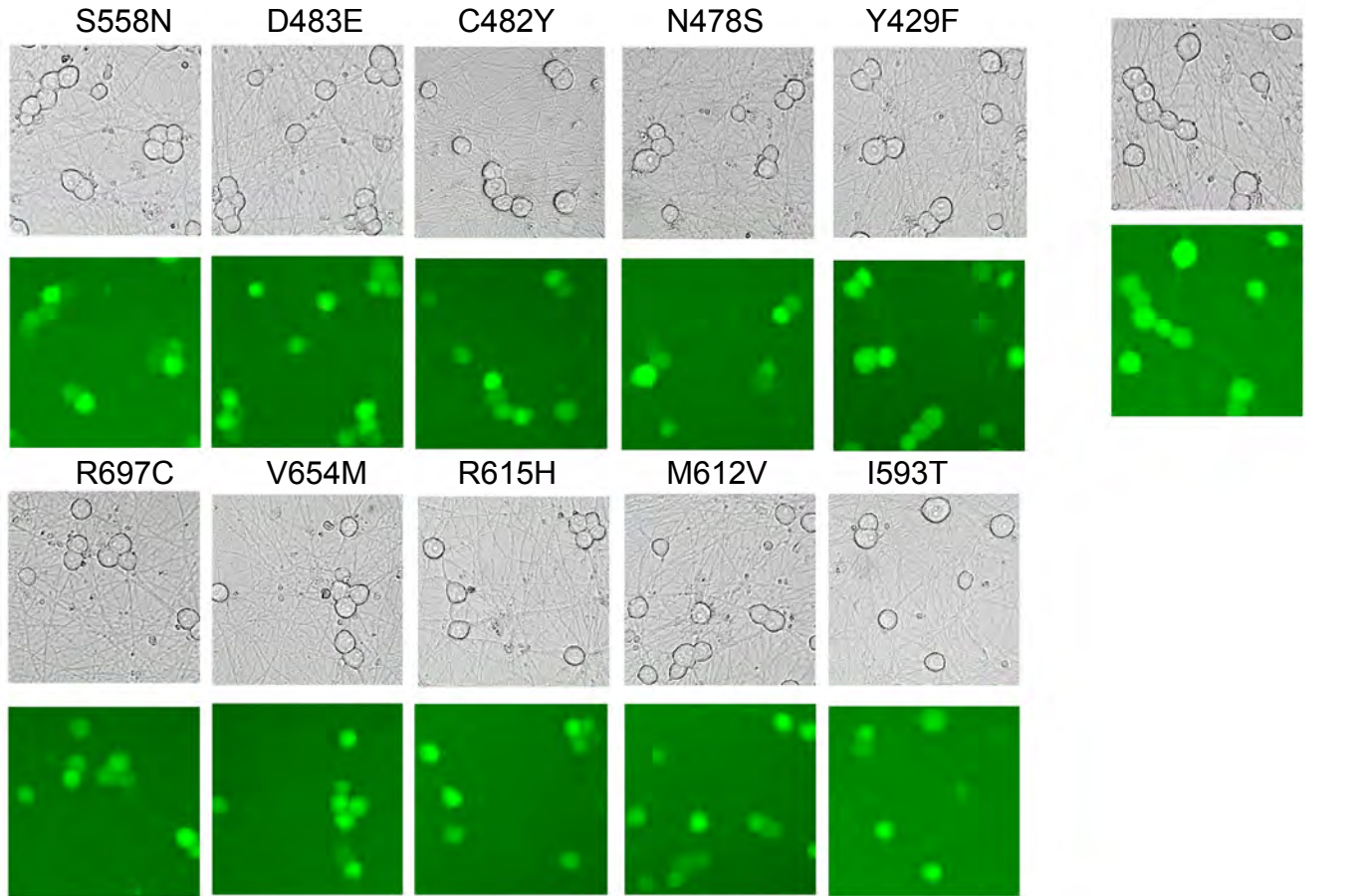

Supplement: Supplementary file 1 — Additional file 1. Bright field and matching fluorescent images of cultured mouse DRG neurons infected with SARM1-EGFP constructs including the human reference allele, every constitutively active SARM1 variant found in ALS patients and ten rare SARM1 variants found in controls. The expression of EGFP in neurons infected with ALS-associated constitutively active SARM1 variants is similar or less than EGFP expression in reference SARM1-infected neurons (compare background fluorescence to cell bodies). [file 13024_2021_511_MOESM1_ESM.pdf]

# EGFP expression in SARM1 construct infected neurons

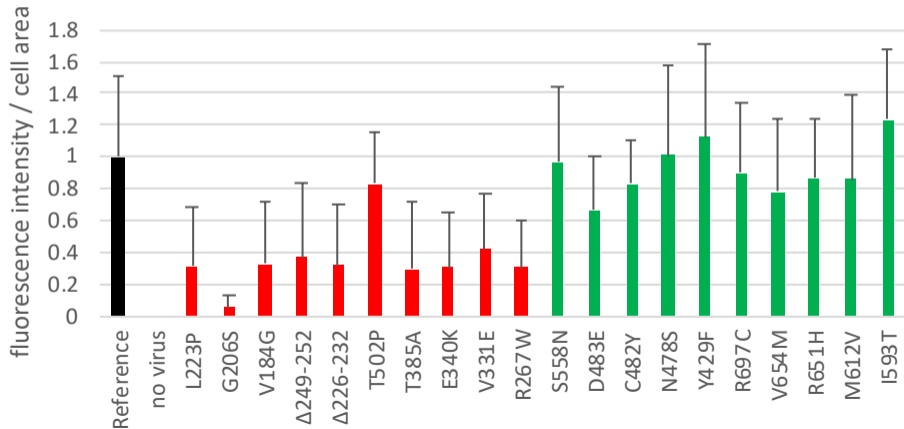

Supplement: Supplementary file 2 — Additional file 2. EGFP fluorescence intensity per cell area in cultured mouse DRG neurons infected with SARM1-EGFP constructs including every constitutively active SARM1 variant found in ALS patients and ten rare SARM1 variants found in controls, normalized to the human reference SARM1 allele. Reduced EGFP expression in cells infected with constitutively active SARM1 alleles is likely due to NAD+ depletion by SARM1 leading to impaired protein translation. [file 13024_2021_511_MOESM2_ESM.pdf]

# cADPR/NAD in CD1 WT neurons

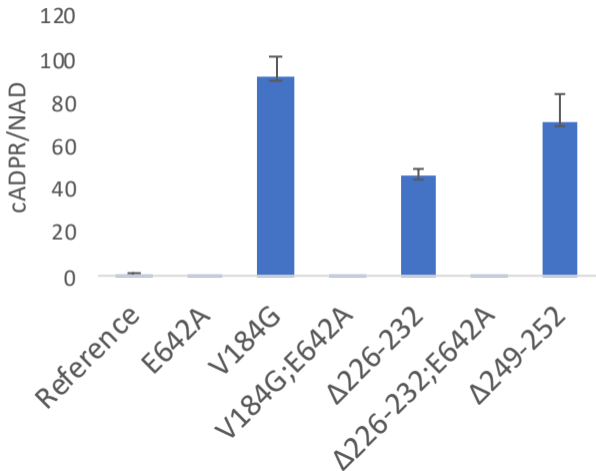

Supplement: Supplementary file 3 — Additional file 3. The ratio of cADPR/NAD+ levels in cultured CD1 wild-type DRG neurons (i.e. expressing endogenous SARM1) infected with human SARM1 constructs carrying rare variants identified in ALS patients. The variants are significantly more active than the reference human SARM1 allele in the presence of endogenous SARM1. This activity is dependent on the activity of the SARM1 mutant, as demonstrated by the loss of activity when a second activity-abolishing mutation, E642A, is introduced into the construct. Data are expressed relative to cADPR/NAD+ in reference SARM1-expressing neurons. [file 13024_2021_511_MOESM3_ESM.pdf]

# Grip strength 3 weeks post-injection

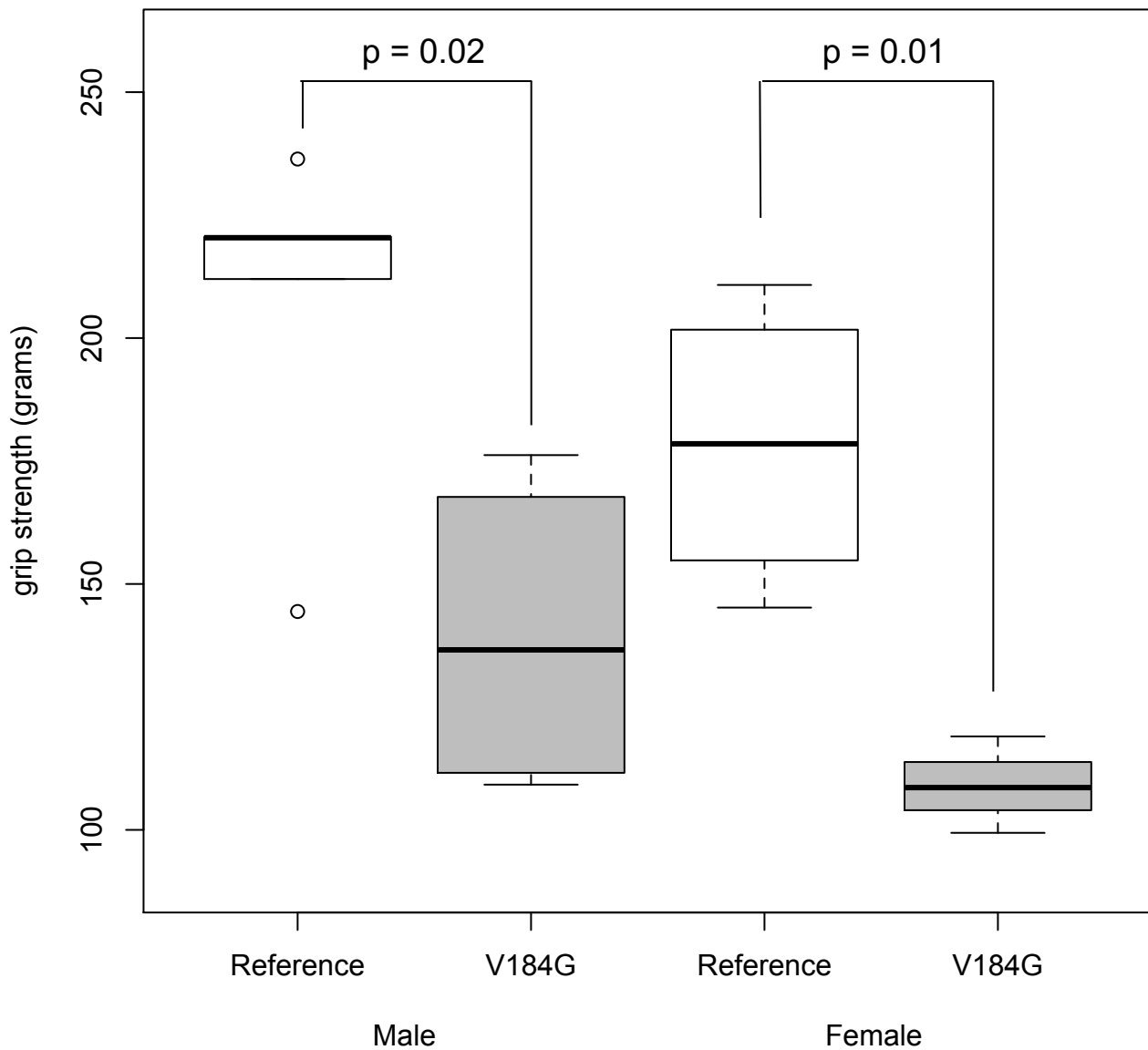

Supplement: Supplementary file 4 — Additional file 4. Forelimb grip strength in male and female mice 3 weeks after injection with SARM1 AAV constructs. [file 13024_2021_511_MOESM4_ESM.pdf]

SARM1<sup>V184G</sup>

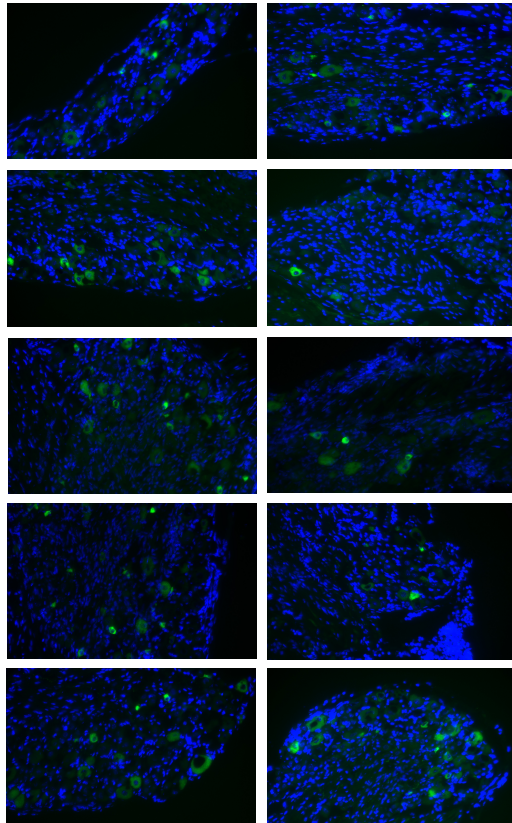

SARM1

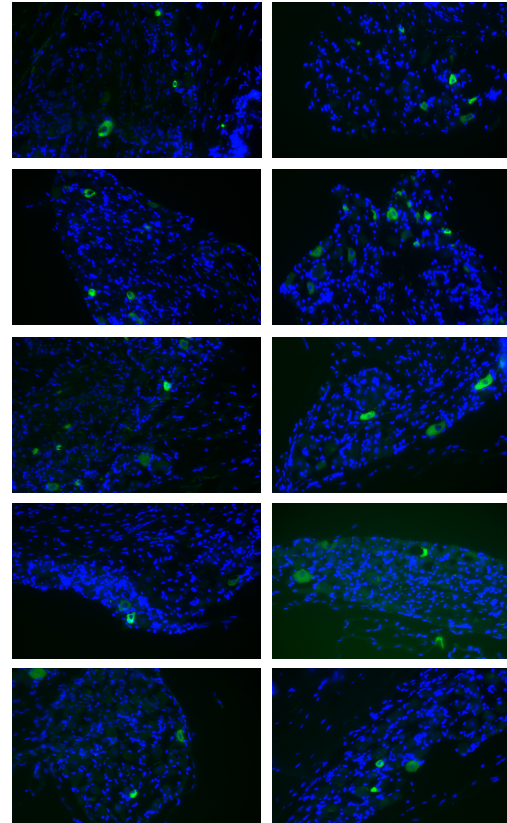

DAPI  
EGFP

Supplement: Supplementary file 6 — Additional file 6. Representative images of dorsal root ganglia from mice 12 weeks post intrathecal injection with AAV constructs containing SARM1 or the ALS-associated constitutively active variant SARM1V184G fused to EGFP, demonstrating similar expression levels from the two constructs. [file 13024_2021_511_MOESM6_ESM.pdf]

# Percent of EGFP-positive DRG neurons in mice 12 weeks after injection with SARM1 AAVs

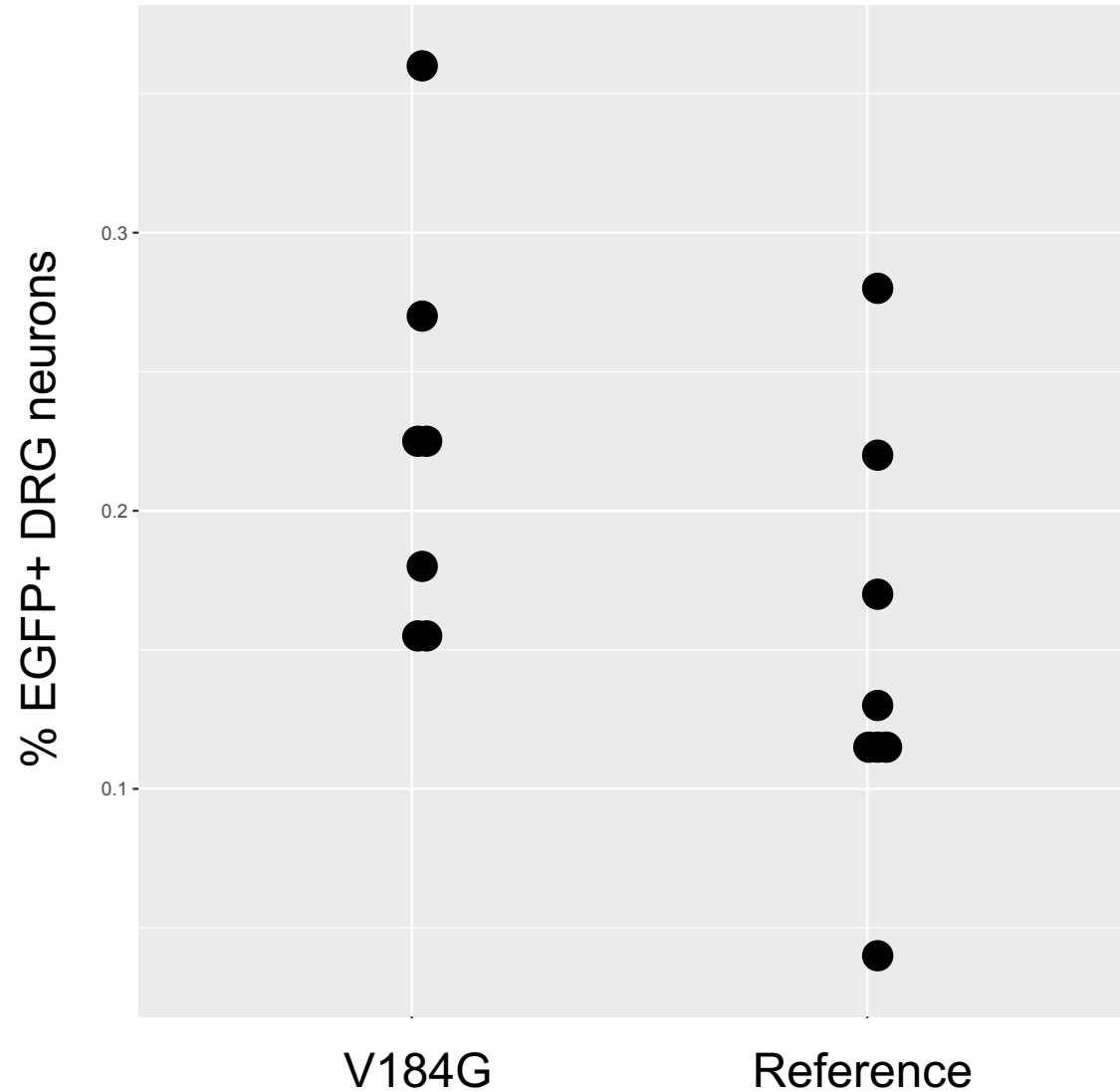

Supplement: Supplementary file 7 — Additional file 7. Percent of EGFP positive dorsal root ganglia from mice intrathecally injected with AAV constructs containing the reference SARM1 allele or SARM1V184G fused to EGFP. [file 13024_2021_511_MOESM7_ESM.pdf]
