# Supplementary material for: Constitutively active SARM1 variants that induce neuropathy are enriched in ALS patients
Source: Mol Neurodegener. 2022 Jan 6;17:1. doi: 10.1186/s13024-021-00511-x (PMC8739729; doi:10.1186/s13024-021-00511-x)
Supplement: Supplementary file 5 — Additional file 5. Percent area of TUNEL staining in spinal cord, and percent area of CD68 staining in sciatic nerves, two days after injecting mice with SARM1 AAV constructs. [file 13024_2021_511_MOESM5_ESM.pdf]

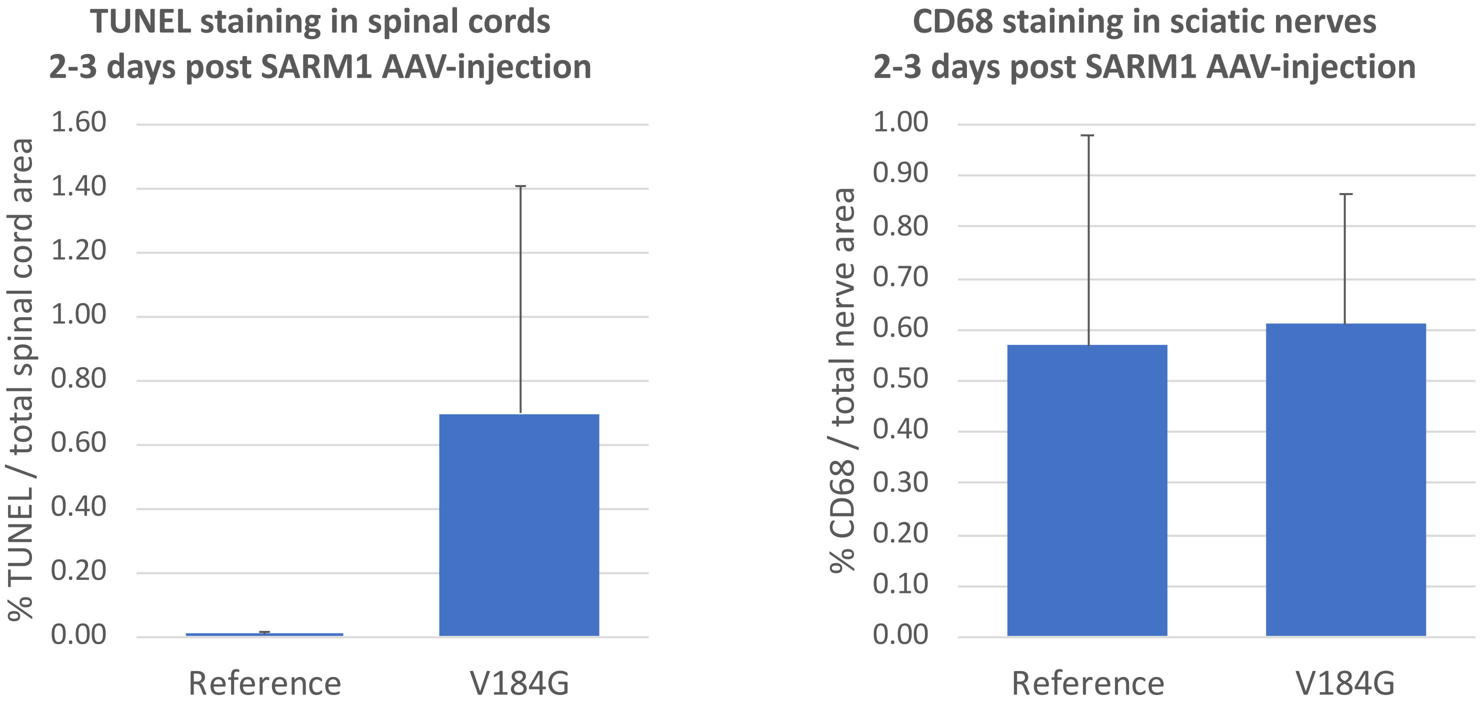

TUNEL staining in spinal cords
2-3 days post SARM1 AAV-injection
% TUNEL / total spinal cord area
1.60
1.40
1.20
1.00
0.80
0.60
0.40
0.20
0.00
Reference
V184G
CD68 staining in sciatic nerves
2-3 days post SARM1 AAV-injection
% CD68 / total nerve area
1.00
0.90
0.80
0.70
0.60
0.50
0.40
0.30
0.20
0.10
0.00
Reference
V184G
